# Influence of Tactical and Situational Variables on Offensive Sequences During Elite European Handball Matches

**DOI:** 10.3389/fpsyg.2022.861263

**Published:** 2022-06-17

**Authors:** Willian Ferrari, Hugo Sarmento, Adilson Marques, Gonçalo Dias, Tiago Sousa, Pedro Antonio Sánchez-Miguel, José Gama, Vasco Vaz

**Affiliations:** ^1^Faculty of Sport Sciences and Physical Education, University of Coimbra, Coimbra, Portugal; ^2^University of Coimbra, Research Unit for Sport and Physical Activity (CIDAF), Faculty of Sport Sciences and Physical Education, Coimbra, Portugal; ^3^Interdisciplinary Centre for the Study of Human Performance (CIPER), Faculty of Human Kinetics, University of Lisbon, Lisbon, Portugal; ^4^ASSERT, Polytechnic Institute of Coimbra, ESEC, Coimbra, Portugal; ^5^Polytechnic Institute of Coimbra, IIA, ROBOCORP, Coimbra, Portugal; ^6^Teacher Training College, University of Extremadura, Cáceres, Spain

**Keywords:** notational analysis, match analysis, goal scoring, performance indicators, handball analysis

## Abstract

The main objective of this study was to analyze the influence of tactical and situational variables on offensive sequences during elite European handball matches. A sample of 55 games and 5.857 offensive sequences from the European Handball Federation Champions League, the selected teams were classified as the top eight teams in the league, were analyzed using *X*^2^ and logistic regression analyses. Results indicated that positional attacks [odds ratio (OR) = 0.34; 95% CI: 0.28–0.42; *p* < 0.001] and fast attacks (OR = 0.46; 95% CI: 0.36–0.57; *p* < 0.001) decreased the probability of success for an offensive sequence by 66% and 54% when compared with counterattacks. Offensive sequences that start in the attacking zone seem to be less effective (~78%) than those that start from a situation of “ball in center.” Additionally, offensive sequences that finished in the defensive zone of the observed team were 3.19 times more effective than those that ended before the 9 m zone. We concluded that compared with offensive sequences where the shot is performed from the 9 m zone, the chances of an offensive sequence ending successfully are 3.65, 3.60, and 2.21 times higher, for offensive sequences where the shot is performed from 9 m, 6 m, and the defensive zone, respectively. On the other hand, many variables seem to impact the performance of handball teams. Nevertheless, a significant challenge remains, and more research needs to be conducted to analyze the obstacles that teams need to overcome while attacking in the most effective way possible.

## Introduction

The modern game of handball was included in the Berlin Olympics in 1936 for the first time after a long period of absence and was introduced again in 1972 at the Munich Olympics (Saavedra, 2018). Today, the sport is played by around 19 million people. Handball involves many actions—offensive actions include throws, passes, jumps, hits, blocks, pushes, runs, and dribbling (Milanese et al., 2012). Given the complexity of this sport, an athlete’s performance in handball depends on multiple factors (Wagner et al., 2014; Almeida et al., 2020). Match analysis in the final of a match helps to evaluate the performance in this specific match, while match analysis after one season, one championship or a tournament cup can contribute to rate and estimate the failure or success of the participating teams and more than this to rate general of that branch of that sport (Bilge, 2012).

In handball, a scored goal is the result of direct and indirect actions, the use of free spaces, and interactions between players. Additionally, several secondary objectives must be achieved during the different phases of the offensive process to achieve the primary objective of scoring a goal (Gruiç et al., 2006; Ferrari et al., 2019). Some of these techniques have been used to investigate the effect of team position type on the effectiveness of the offensive process (Büchel et al., 2019). Offensive tactical activity is a crucial feature of team sports and can be defined as the planned and premeditated management of all offensive systems with the ultimate goal of scoring points (Rogulj et al., 2004).

Performance analysis in team sports has been part of the agenda of sports scientists for some time now (Sarmento et al., 2011, 2018a,b; Ferrari et al., 2019; Konefał et al., 2019; Clemente et al., 2020), and handball is no exception. Recently, researchers have begun to apply increasingly sophisticated statistical procedures (Almeida et al., 2020) to data sets to understand the factors that promote or hinder offensive effectiveness in handball. Such procedures include network analysis (Korte and Lames, 2019), the classification tree approach (Saavedra et al., 2019), cluster analysis (Gómez-López et al., 2019), polar coordinate analysis (Antonio et al., 2019), and t-patterns analysis (Pic, 2018).

The observation and analysis of sports have evolved considerably over the past few years due to technological advances. Naturally, the actions that most commonly lead to goals (the main objective of this sport) have been the focus of researchers’ attention (Rogulj, 2000; Vuleta et al., 2005; Rogulj et al., 2011; Ferrari et al., 2019; Hatzimanouil, 2019; Zapardiel et al., 2019). This trend is seen in many sports such as football (Armatas et al., 2007) and futsal (Sarmento et al., 2016). However, the offensive process in handball has received relatively little attention from the scientific community (Ferrari et al., 2019).

Nevertheless, available research on male handball provides some useful insights. For example, research has shown that the higher number of goals scored in the second half of games seems to be related to the fatigue experienced by players (Povoas et al., 2014; Büchel et al., 2019) as well as a decrease in the intensity of defensive actions (Skarbalius et al., 2013). Additionally, goals are commonly scored during the last 5 min of each match half (Oliveira et al., 2012) and after time-outs requested by coaches (Prieto et al., 2016).

Furthermore, several researchers have investigated the influence of tactical variables on the efficacy of the offensive process in handball. Rogulj and Srhoj (2009) verify the influence of the elements of collective attack tactics on the game’s outcome. Rogulj et al. (2011) conclude the performance of collective tactics in an attack recognized as efficient in competitive conditions should be based on the performance of as many as possible fast attacks on an unprepared defense and on short position attacks. Alexandru and Acsinte (2017) allowed us to know the evolution recorded by each team, regarding the frequency of the scoring chances, the frequency of goals, the effectiveness, and the differences between the two championship, observed the tendencies to improve their play.

Foreti et al. (2013) the research findings to establish quantitative contributions of situational activities of playing positions and game phases to the final match result, and Prieto et al. (2016) analyzing the scoring processes coordination of the teams can provide highly valuable information for a better understanding of the dynamics of handball games. Dumangane et al. (2009) concluded that the probability of scoring a goal does not depend directly on the past performance of one’s team. Instead, it depends indirectly on the past performance of the opposing team and the difference between the teams’ scores during the last ball possession. Additionally, Rogulj et al. (2004, 2011) and Rogulj and Srhoj (2009) concluded that teams that made continuous short-term attacks against unorganized defenses, as well as short positional attacks (less than 25 s), were more likely to succeed than other teams.

Despite the importance of situational variables on team performance, only a few studies have comprehensively examined such variables (Prieto et al., 2015; Ferrari et al., 2019). To provide a more comprehensive and generalized view on recent handball tactics and their associated effectiveness, it is important to use additional new variables such as field zones, offensive actions, match status and shooting zones, taking into account the high-level club championship. In addition, understanding how contextual factors influence performance could improve the quality of research in match analysis. Therefore, the aim of this study is to examine the influence of tactical and situational variables on the success offensive sequences during elite European handball matches.

## Materials and Methods

### Sample

A sample of 55 games and 5.857 offensive sequences from the European Handball Federation (EHF) Champions League (from 2012/2013 to 2016/2017) were analyzed. The selected teams were classified as the top eight teams in the league based on their final rankings. Matches that ended in a tie were excluded from the analysis. The study was conducted according to the guidelines of the Declaration of Helsinki, University of Coimbra and the CAPES—Brazilian Federal Agency for Support and Evaluation of Graduate Education within the Ministry of Education of Brazil (approval number: 00835/2014-05).

### Data Coding System

**Figure 1:**
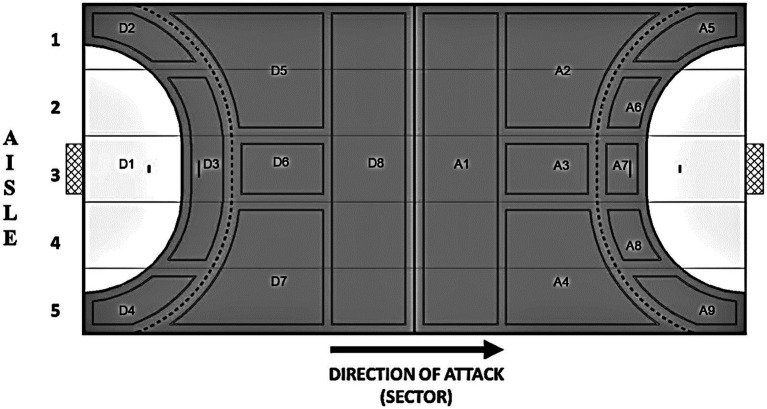
Field zones divided into 17 zones and five aisles with the numbering of zones designated according to the direction of the attack (Ferrari et al., 2020) D, Defense and A, Attack.

Data were analyzed using a specific notational system that was developed and validated by Ferrari et al., (2018). This system combines pitch zones and key offensive activities and subcategorizes them into (1) team possession type (Positional attack; Fast attack; and Counterattack); (2) situational variables (Type of offensive actions, Match half, Match status, Match outcome, Numerical relationship, and Interaction context); and (3) starting (Goalkeeper, Defense, Attack, and Ball in center), (4) shooting (9, 9–6, 6, and 7 m, and Defense), and (5) finishing zones (Before 9 m, between 9 and 6 m, and Defense zone; Figure 1; Tables 1, 2).

**Table 1:** Description of variables and definitions of category used in the team match performance analysis (Team Possession Type, Type of offensive actions, Match half, Match status, Match outcome, and Numerical relationship).

Criteria	Categories	Definition
Team possession type	Positional attack	An action is considered when each player occupies their specific position and initiates interactions to move the defense, this phase begins when the opponent’s defense is established in their position, against an organized offensive system
	Fast attack	Is considered as a second offensive chance, made by later players in the defensive system, who progressed in the field with speed, through quick passes to the attack, in order to create a situation of superiority or defensive disorganization of the attack to opponents’ team.
	Counterattack	This offensive method starts in the defensive field, trying to get as fast as possible to the opponents’ goal with as few passes as possible.
Type of offensive actions	Complete offensive sequences (OS)	Complete collective actions (e.g., start, progression, and completion) are those that result from dynamic or static play, implying a start, a progression development in the field of play for more offensive areas and a finalization of the offensive sequence (with or without efficiency).
	Set pieces	Actions that start by a stopped ball situation (e.g., 7 m penalty shot, direct or indirect free kick, foul, etc.) that imply a short finalization and imply a rapid finalization of the offensive process (<3 passes between the players).
Match half	First half	From the referee’s whistle the beginning of the first half to the whistle at the end of this part.
	Second half	From the referee’s whistle the beginning of the second half to the whistle at the end of this part.
Match status		Is the current score in which the analyzed action is detected.
	Losing >5	The observed team has at least 5 or more goals in disadvantage to the opponent
	Losing >3	The observed team has at least 3 or 4 goals in disadvantage to the opponent
	Losing >1	The observed team has at least 1 or 2 goals in disadvantage to the opponent
	Drawing	The team observed is tied in goals with the opponent
	Winning >1	The observed team has at least 1 or 2 goals made to advantage to the opponent
	Winning >3	The observed team has at least 3 or 4 goals made to advantage to the opponent
	Winning >5	The observed team has at least 5 or more goals made to advantage to the opponent
Match outcome		Is the final score of the game.
	Losing >5	The observed team lost the game by 5 or more goals at a disadvantage to the opponent
	Losing >3	The observed team lost the game by 3 or 4 goals in disadvantage to the opponent
	Losing >1	The observed team lost the game by 1 or 2 goals at a disadvantage to the opponent
	Winning >1	The observed team won the game by at least 1 or 2 goals in advantage to the opponent
	Winning >3	The observed team won the game by at least 3 or 4 goals in advantage to the opponent
	Winning >5	The observed team won the game for at least 5 or more goals in advantage to the opponent

**Table 2:** Description of variables and definitions of category used in the team match performance analysis (starting, shooting and finishing zones, effectiveness, and interaction context).

Criteria	Categories	Definition
Starting zones	Goalkeeper	The team starts the offensive process behind a goalkeeper’s defense or a shot outside the goalpost.
	Defense	It is characterized by a defensive rebound of the goalkeeper, an interception of the defenders or a foul or technical failure of the opposing team, and the team thus of the beginning with the ball in the zone of the defense.
	Attack	Starts when the team manages to recover a ball still in the attacking field.
	Ball in center	The offensive process starts after the team suffers a goal or start of the first or second part of the game.
Finishing zones	Before 9 m	Any action that was completed before the dashed line of the 9 m represented in Figure 1 (A1, A2, A3, and A4).
	Between 9 and 6 m	Any offensive action that was completed after the 9-m dashed line represented in Figure 1 (A5, A6, A7, A8, and A9).
	Defense zone	Any action of the offensive process that has been completed in the zone of defense represented in the figure as all zones containing “D.”
Shooting zones	9 m	The player making the shot has his last support foot placed before the dashed line.
	9–6 m	The player who hit the ball had his support foot touching the ground, between the dashed line (9 m) and the 6 m.
	6 m	The player, with his jump, invades the airspace of the area, where he had to finish before landing.
	7 m	Was carried out while 7 m penalty shot was awarded.
	Defense	When the shot was taken from the field of defense of the team.
Numerical relationship	Equality	Action in which the teams are equal in number of players in the field.
	Inferiority	Action in which the teams are in numerical inferiority of players in the field.
	Superiority	Action in which the teams are in numerical superiority of players in the field.
Effectiveness	With effectiveness	Shot with a goal scored.
	Without effectiveness	Recovery of ball possession by the opponent, ball out, violation of the rules of the game, shot defended by the goalkeeper, shot out, shot into the opponent.
Interaction context	Time of duration	Total time from the beginning of the offensive action to the final offensive action.
	Total of passes	Number of interactions between players from the beginning to the conclusion.

### Statistical Analyses

All analyses were performed using statistical software (IBM SPSS, version 24.0). Intra-observer and inter-observer agreement (Table 3) were quantified using Cohen’s kappa (Cohen, 1960). For the statistical analysis, a logistic regression analysis was performed to examine the independent and interactive effects of all independent variables. The statistical model employed involved reverse hierarchical elimination (Kleinbaum et al., 2002). The level of statistical significance was set at 0.05 was used for all statistical tests.

**Table 3:** Kappa values for intraobserver and interobserver.

Category	Intraobserver		Interobserver	
	Kappa	CI (95%)	Kappa	CI (95%)
Team possession type	0.92	0.91–0.94	0.91	0.88–0.93
Type of offensive actions	0.93	0.92–0.95	0.90	0.87–0.92
Match half	0.99	0.98–0.99	0.99	0.98–0.99
Match status	0.99	0.98–0.99	0.99	0.98–0.99
Match outcome	0.99	0.98–0.99	0.99	0.98–0.99
Starting zones	0.88	0.86–0.90	0.89	0.86–0.91
Finishing zones	0.89	0.87–0.91	0.88	0.86–0.90
Shooting zones	0.90	0.88–0.92	0.90	0.87–0.92
Numerical relationship	0.91	0.89–0.93	0.90	0.87–0.92
Effectiveness	0.99	0.98–0.99	0.99	0.98–0.99
Interaction context	0.89	0.87–0.91	0.87	0.85–0.89

Two handball analysts experienced in match analysis procedures used this observational instrument tool to analyze offensive sequences. After a training period (1 week), each analyst analyzed six randomly selected games (corresponding to 10.9% of the sample). Based on their analyses, intra observer reliability was assessed using the offensive sequences of the same six games. The lead author of this study then repeated these on two occasions 4 weeks later.

## Results

A total of 5.857 offensive sequences were analyzed (52.24% with effectiveness) in the following subsets: (1) positional attacks (*n* = 4,428), fast attacks (*n* = 854), counterattacks (*n* = 575). Differences were observed in the probability of producing effective offensive sequences for all variables except for the main variable of “match status” (Table 4).

**Table 4:** Differences in possession outcome according to team position type and situational variables.

Categories	Without effectiveness, *n* (%)	With effectiveness, *n* (%)	OR (95% CI)	*p*
**Team possession type**				0.001^**^
Counterattack	152 (26.4)	423 (73.6)	1.00 (ref.)	
Positional attack	2,268 (51.2)	2,160 (48.8)	0.34 (0.28–0.42)	0.001^**^
Fast attack	377 (44.1)	477 (55.9)	0.46 (0.36–0.57)	0.001^**^
**Type of offensive actions**				0.001^**^
Complete offensive sequence	1,340 (34.8)	2,510 (65.2)	1.00 (ref.)	
Set pieces	356 (39.3)	550 (60.7)	0.78 (0.61–0.98)	0.037^*^
**Match half**				0.334
First half	1,378 (48.4)	1,468 (51.6)	1.00 (ref.)	
Second half	1,387 (47.2)	1,553 (52.8)	1.00 (0.86–1.16)	0.964
**Match status**				0.249
Drawing	485 (49.4)	497 (50.6)	1.00 (ref.)	
Losing > 1	365 (48.5)	387 (51.5)	1.03 (0.79–1.34)	0.851
Losing > 3	634 (47.2)	709 (52.8)	1.15 (0.91–1.47)	0.242
Losing > 5	264 (47.1)	297 (52.9)	1.67 (1.20–2.33)	0.002^**^
Winning > 1	297 (45.2)	360 (54.8)	1.06 (0.80–1.40)	0.698
Winning > 3	595 (49.6)	604 (50.4)	0.81 (0.63–1.05)	0.108
Winning > 5	157 (43.3)	206 (56.7)	0.66 (0.44–0.99)	0.043^*^
**Match outcome**				0.001^**^
Losing > 1	256 (47.7)	281 (52.3)	1.00 (ref.)	
Losing > 3	683 (49.7)	690 (50.3)	0.67 (0.51–0.90)	0.007^**^
Losing > 5	568 (55.8)	450 (44.2)	0.54 (0.39–0.75)	0.000^**^
Winning > 1	249 (46.4)	288 (53.6)	0.78 (0.55–1.09)	0.144
Winning > 3	571 (46.3)	661 (53.7)	1.04 (0.78–1.38)	0.806
Winning > 5	470 (40.5)	690 (59.5)	1.50 (1.11–2.03)	0.008^**^

* *p < 0.05*;

** *p < 0.01*.

Differences were also found between the different “types of attack” in terms of whether they produced an effective offensive sequence. In this sense, positional attacks [odds ratio (OR) = 0.34; 95% CI: 0.28–0.42; *p* < 0.001] and fast attacks (OR = 0.46; 95% CI: 0.36–0.57; *p* < 0.001) decreased the probability of success by 66% and 54% when compared with counterattacks.

Additionally, the type of offensive sequence seems to influence the efficacy. Elaborate offensive sequences tend to be more effective than short ones that result from a direct/indirect free kick or a 7 m penalty shot (OR = 0.78; 95% CI: 0.61–0.98; *p* = 0.043).

Furthermore, teams losing by more than five goals were 1.67 times more likely (95% CI: 1.20–2.33; *p* < 0.002) to perform a successful offensive sequence when compared with teams losing by more than one goal. Also, teams winning by more than five goals had a 44% weaker chance (OR = 0.66; 95% CI: 0.44–0.99; *p* < 0.001) of performing a successful offensive sequence when compared with teams losing by more than one goal.

Additionally, the probability of producing an effective offensive sequence is 1.5 times higher (95% CI: 1.11–2.03; *p* = 0.008) for a team that is winning a game by more than five goals when compared to a team that is losing by one or two goals. When a team is losing by more than 3 or more than five goals, their likelihood of performing a successful offensive sequence reduces by 33% (OR = 0.67; 95% CI: 0.51–0.90; *p* = 0.007) and 46% (OR = 0.54; 95% CI: 0.39–0.75; *p* < 0.001), respectively, when compared with teams losing by more than one goal.

Additional differences were observed regarding the odds ratio for producing effective offensive sequences based on the zone of the field where the offensive process starts. Offensive sequences that started from the attacking zones were less effective (~78%) than those that started from a situation of “ball in center.” Furthermore, the results revealed that offensive sequences that finished in the defensive zone of the observed team were 3.19 times more effective (95% CI: 1.94–5.25; *p* < 0.001) than those that ended before the 9 m zone (offensive midfielder; Table 5).

**Table 5:** Differences in possession outcome according to start, finishing and shooting zones, and numerical relationship.

Categories	Without effectiveness, *n* (%)	With effectiveness, *n* (%)	OR (95% CI)	*p*
**Starting zone**				0.001^**^
Goalkeeper	601 (48.0)	652 (52.0)	0.94 (0.78–1.14)	0.527
Defense	579 (40.7)	845 (59.3)	1.08 (0.89–1.33)	0.429
Attack	4 (66.7)	2 (33.3)	0.22 (0.04–1.29)	0.093
Ball in center	1,613 (50.8)	1,561 (49.2)	1.00 (ref.)	
**Finishing zones**				0.001^**^
Before 9 m	1,051 (65.8)	548 (34.2)	1.00 (ref.)	
Between 9 and 6 m	1,696 (40.5)	2,491 (59.5)	1.13 (0.68–1.87)	0.637
Defense zone	50 (68.5)	23 (31.5)	3.19 (1.94–5.25)	0.001^**^
**Shooting zones**				0.001^**^
9 m	628 (53.6)	544 (46.4)	1.00 (ref.)	
9–6 m	421 (44.8)	519 (55.2)	1.42 (1.20–1.69)	0.001^**^
6 m	530 (24.3)	1,655 (75.7)	3.60 (3.10–4.19)	0.001^**^
7 m	101 (24.0)	319 (76.0)	3.65 (2.84–4.69)	0.001^**^
Defense	12 (34.3)	23 (65.7)	2.21 (1.09–4.49)	0.028^*^
**Numerical relationship**				0.001^**^
Equality	2,297 (47.9)	2,503 (52.1)	1.00 (ref.)	
Inferiority	193 (59.8)	130 (40.2)	0.64 (0.47–0.87)	0.004^**^
Superiority	307 (41.8)	427 (58.2)	1.38 (1.09–1.75)	0.007^**^

* *p < 0.05*;

** *p < 0.01*.

Differences in offensive sequences were also found based on the finishing zone of the field. When compared with offensive sequences in which the shot was performed from 9 m zone, the chance of an offensive sequence ending successfully is 3.65 higher (95% CI: 2.84–4.69; *p* < 0.001), 3.60 times higher (95% CI: 3.10–4.19; *p* < 0.001), and 2.21 times higher (95% CI: 1.09–4.49; *p* = 0.028) when the shot is performed from 9 m, 6 m, and defensive zones, respectively.

For the main variable of “numerical relationship,” the chances of an offensive sequence ending successfully is 1.38 times higher when the observed team has numerical superiority (95% CI: 1.09–1.75; *p* = 0.007), whereas having fewer players on the field decreases the chances of performing a successful offensive sequence by 36% (95% CI: 0.47–0.87; *p* = 0.004).

The odds ratios presented indicate that a one-second increase in the duration of an offensive sequence causes a 1% decrease (OR = 0.99; 95% CI: 0.98–0.99; *p* < 0.001) in the probability that the outcome will be successful. Additionally, an extra pass increases the probability of success (OR = 1.03; 95% CI: 1.01–1.05; *p* = 0.008; Table 6).

**Table 6:** Differences in possession outcome according the duration and total of passes.

Categories	Without goal, *n* (%)	With Goal, *n* (%)	OR (95% CI)	*p*
Time of duration	36.35 (35.58–37.13)	32.33 (31.57–33.08)	0.99 (0.98–0.99)	0.001^**^
Total of passes	13.10 (12.82–13.37)	11.66 (11.39–11.94)	1.03 (1.01–1.05)	0.008^**^

** *p < 0.01*.

## Discussion

To the best of our knowledge, this is the first study to explore the combined effects of tactics and situational factors concerning offensive effectiveness among teams in the EHF Champions League. Regarding tactics, elite handball teams playing mostly in positional attacks (Rogulj et al., 2004; Foreti et al., 2013; Gutiérrez Aguilar and Ruiz, 2013). In terms of team possession type, differences in the probability of efficacy depended on the specificities of offensive sequences. The traditional debate about what style of play is most effective has been debated by researchers in handball and other sports for a long time (Ruiz Sánchez et al., 2017; Sarmento et al., 2018b). Counterattacks seem to be the offensive sequences that have the most efficacy in handball, as is the case in other sports, such as football. The “fast type” of attacks (counterattacks and positional attacks) appear to promote success due to their sudden execution and the quick transition from defending to attacking while the opponent’s defense is unbalanced (Rogulj et al., 2004; Dumangane et al., 2009; Ruiz Sánchez et al., 2017).

Nevertheless, our results reveal that elaborate offensive sequences (i.e., those resulting from positional attacks, counterattacks, or fast attacks) seem to be more effective than shorter sequences (e.g., situations resulting from a dead ball; those involving no more than three passes; or those resulting from a 7 m penalty shot, a 9 m direct/indirect free kick, etc.). Although this might seem to contradict with the previous results, this situation is plausible because in situations that start with a dead ball, the opposing defense can organize itself to prevent a successful attack. As stated in previous research, these types of situations significantly influence the outcomes of matches.

Concerning situational variables match status has a direct influence on the probability that a team will end an offensive sequence by scoring a goal. Teams losing by more than five goals had a 1.67 times greater chance of performing a successful offensive sequence than teams losing by more than one goal. Conversely, teams winning by more than five goals were 44% less likely to perform a successful offensive sequence than teams losing by more than one goal. This could be the case because teams slow their pace when they are winning by several goals (Debanne and Laffaye, 2015; Molfetas et al., 2019), as they tend to focus more on maintaining their advantage than increasing it (Dumangane et al., 2009). Sometimes, having a comfortable advantage in a match can increase the confidence of the leading team; however, this can lead to overconfidence, which causes some devolution among the leading team and favors the efficiency of the trailing team (Schinke et al., 2018).

Additionally, the zones of the field in which an offensive sequence starts seems to influence the sequence’s outcome. Offensive sequences that start in an attacking zone are less effective (~78%) than those that start in a situation of “ball in center.” As is the case with offensive actions resulting from dead balls, this result could be because the opponent has an opportunity to organize their defense before the sequence begins. The quality of transferring from the position defense into transition attack depends on the speed of returning the ball back in the game by the goalkeeper (Burger et al., 2013). However, these data should be analyzed with some caution due to the small number of occurrences recorded.

The main categories of interest (“finishing zones”) did not influence the zone of the field where the offensive sequence ends in the offensive midfield (before 9 vs. 6–9 m) relative to their efficacy. This result was unexpected because the previous research indicates that offensive sequences that end in zones closer to the goal have greater efficacy (Volossovitch and Ferreira, 2003; Hatzimanouil, 2019).

Concerning shooting zones, our results confirmed that 7 m penalty shots have the highest level of efficacy. Previous studies demonstrate that a team’s effectiveness in these types of situations is crucial to match outcomes (Vuleta et al., 2012; Daza et al., 2017). Excluding 7 m penalty shots, the most effective zone for finishing an offensive sequence seems to be the 6 m zone. The efficacy of teams in these situations is also considered as a meaningful performance indicator that distinguishes winning teams from losing teams in balanced game contexts (Srhoj et al., 2001; Gruiç et al., 2006; Foretić et al., 2010; Meletakos et al., 2011; Teles and Volossovitch, 2015).

The difference between shots taken from the 6 m zone and shots taken from the 9 m zone in terms of their efficacy has not been studied in depth prior to the present work. The data presented in this study are significant, considering that most of the offensive sequences that end in a shot in handball end in these zones. This study’s differentiated analysis of the areas where shots are taken from has been necessary for a long time due to changes in the rules of the game (Skarbalius and Krusinskiene, 2003; Ferrari et al., 2018). A particularly interesting result emerged from the analysis, shots performed from the defensive zone that provide a chance for an offensive sequence were 2.21 times more likely to succeed than offensive sequences in which the shot is performed from the 9 m zone. This result reflects the effectiveness of teams in taking advantage of the temporary defensive disorganization of the opposing team, especially when the team is playing without their goalkeeper in the goal.

Concerning the numerical relationship between teams, we concluded that the chance of an offensive sequence ending successfully is 1.38 times higher when the observed team has a numerical advantage, whereas having fewer players on the field leads to a 36% decrease in the success rate of offensive sequences. In this sense, there seems to be a clear benefit to the teams that take advantage of situations when they have numerical superiority due to a 2-min sanction imposed on a player of the opposing team (Fasold and Redlich, 2018; Gryko et al., 2018).

Finally, the multiple regression analysis revealed that long possessions (in terms of the number of the passes performed) were more effective than short possessions. Specifically, an extra pass increases the probability that the offensive sequence will be successful by 1.03 times. However, this is only true when the extra pass does not increase the duration of the offensive sequence, as a one-second increase in the duration of an offensive sequence decreases the probability of success by 1%. These results corroborate previous findings in the context of handball (Gutiérrez Aguilar and López, 2010; Ferrari et al., 2016; Ferreira et al., 2018; Sarmento et al., 2018b).

This study provides practical implications to coaches, players, and sports scientists in designing specific training situations that improve the effectiveness of the offensive process in handball matches. A possible limitation of this study is associated with not having considered the different movements within the positional attack process, and not having analyzed the type of defense used by the opposing teams at the moment of data collection. Additionally, our results are specific to the sample that we used and not transferable to other studies. Future research should focus on larger and specific groups of actions and the effectiveness of the defensive process in handball matches.

## Conclusion

The results of this study provide valuable information due to the inclusion of new categories that had been unexplored by researchers, exhibiting the increased effectiveness of shots between 9 and 6 m, shots in the defensive field being a new factor to be explored because of the changing rules of the game. The fast attack and its effectiveness mainly in the counter goal is increasingly being used by teams, showing to be an evolution in the handball game leaving increasingly fast with offensive actions of short duration and the fewest possible passes, preventing opposing teams to have their defense organized.

According to the data obtained there are many variables that seems to impact the performance of handball teams. From our results, we highlighted the high effectiveness in counter-attacking shots and shots at 6 m and 7 m penalties. However, a significant challenge remains, and further research is needed to analyze the obstacles that teams need to overcome to more accurately reach the main goal which is the goal.

## Data Availability Statement

The raw data supporting the conclusions of this article will be made available by the authors, without undue reservation.

## Ethics Statement

The study was reviewed and approved by the University of Coimbra and the CAPES—Brazilian Federal Agency for Support and Evaluation of Graduate Education within the Ministry of Education of Brazil (approval number: 00835/2014-05). The study was conducted according to the guidelines of the Declaration of Helsinki. Written informed consent was obtained from all participants involved in the study.

## Author Contributions

WF, HS, and VV contributed to the conception and designed the research study. WF conceived the data collection. WF, HS, GD, TS, and VV analysis and interpretation the data. WF, HS, AM, GD, TS, PS-M, JG, and VV performed the drafting the article and/or its critical revision. All authors contributed to the editorial changes in the manuscript. All authors contributed to the article and approved the submitted version.

## Funding

This work is funded by CAPES—Brazilian Federal Agency for Support and Evaluation of Graduate Education within the Ministry of Education of Brazil.

## Conflict of Interest

The authors declare that the research was conducted in the absence of any commercial or financial relationships that could be construed as a potential conflict of interest.

## Publisher’s Note

All claims expressed in this article are solely those of the authors and do not necessarily represent those of their affiliated organizations, or those of the publisher, the editors and the reviewers. Any product that may be evaluated in this article, or claim that may be made by its manufacturer, is not guaranteed or endorsed by the publisher.

## References

[ref1] Alexandru E. Acsinte A. (2017). The particularized evolution, for the national teams, of the scoring chances in team handball. Gymnasium 12, 158–172.

[ref2] Almeida A. G. Merlin M. Pinto A. Torres R. D. S. Cunha S. A. (2020). Performance-level indicators of male elite handball teams. Int. J. Perform. Anal. Sport 20, 1–9. doi: 10.1080/24748668.2019.1694305

[ref3] Antonio J. Diz V. Morillo Baro J. P. Reigal R. E. Morales-Sánchez V. Hernández-Mendo A. (2019). Contextual factors and decision-making in the behaviour of finalization in the positional attack in beach handball: diferences by gender through polar coordinates analysis. Front. Psychol. 10:1386. doi: 10.3389/fpsyg.2019.01386, PMID: 31263442 PMC6585176

[ref4] Armatas V. Yiannakos A. Sileloglou P. (2007). Relationship between time and goal scoring in soccer games: analysis of three World Cups. Int. J. Perform. Anal. Sport 7, 48–58. doi: 10.1080/24748668.2007.11868396

[ref5] Bilge M. (2012). Game analysis of Olympic, World and European Championships in men’s handball. J. Hum. Kinet. 35, 109–118. doi: 10.2478/v10078-012-0084-7, PMID: 23486176 PMC3588687

[ref6] Büchel D. Jakobsmeyer R. Döring M. Adams M. Rückert U. Baumeister J. (2019). Effect of playing position and time on-court on activity profiles in german elite team handball. Int. J. Perform. Anal. Sport. 19, 832–844. doi: 10.1080/24748668.2019.1663071

[ref7] Burger A. Rogulj N. Foretić N. Čavala M. (2013). Analysis of rebounded balls in a team handball match. SportLogia 9, 53–58. doi: 10.1080/24748668.2007.11868396

[ref8] Clemente F. M. Sarmento H. Aquino R. (2020). Player position relationships with centrality in the passing network of world cup soccer teams: win/loss match comparisons. Chaos, Solitons Fractals 133:109625. doi: 10.1016/j.chaos.2020.109625

[ref9] Cohen J. (1960). A coefficient of agreement for nominal scales. Educ. Psychol. Meas. 20, 37–46. doi: 10.1177/001316446002000104, PMID: 34968881

[ref10] Daza G. Andrés A. Tarragó R. (2017). Match statistics as predictors of team’s performance in elite competitive handball. RICYDE. Rev. Int. Cienc. Deporte 13, 149–161. doi: 10.5232/ricyde2017.04805

[ref11] Debanne T. Laffaye G. (2015). Motivational cues predict the defensive system in team handball: a model based on regulatory focus theory. Scand. J. Med. Sci. Sports 25, 558–567. doi: 10.1111/sms.12328, PMID: 25262855

[ref12] Dumangane M. Rosati N. Volossovitch A. (2009). Departure from independence and stationarity in a handball match. J. Appl. Stat. 36, 723–741. doi: 10.1080/02664760802499329

[ref13] Fasold F. Redlich D. (2018). Foul or no foul? Effects of permitted fouls on the defence performance in team handball. J. Hum. Kinet. 63, 53–59. doi: 10.2478/hukin-2018-0006, PMID: 30279941 PMC6162971

[ref14] Ferrari W. Dias G. Sousa T. Sarmento H. Vaz V. (2020). Comparative analysis of the offensive effectiveness in winner and losing handball teams. Front. Psychol. 11:547110. doi: 10.3389/fpsyg.2020.547110, PMID: 33071863 PMC7544740

[ref15] Ferrari W. Sarmento H. Vaz V. (2019). Match analysis in handball: a systematic review. Montenegrin J. Sports Sci. 8, 63–76. doi: 10.26773/mjssm.190909, PMID: 34052518

[ref16] Ferrari W. Vaz V. Dias G. Gama J. Sousa T. Couceiro M. (2016). “Conexão e interação no Andebol” in Abordagem das networks no desporto: Fundamentos e aplicações práticas (Coimbra, Portugal: Faculdade de Ciências do Desporto e Educação Física. Universidade de Coimbra), 75–94.

[ref17] Ferrari W. Vaz V. Sousa T. Couceiro M. Dias G. (2018). Development and validation of a notational instrument to study the offensive process in handball. J. Sports Pedagog. Phys. Educ. 4, 27–34.

[ref19] Ferreira A. Graça A. Estriga M. L. Cruz E. (2018). O impacto de uma abordagem compreensiva de curta duração sobre o desempenho das ações ofensivas no jogo de andebol. E-balonmano. com: Journal of Sports Science, 14, 35–44.

[ref20] Foreti N. Rogulj N. Papi V. (2013). Empirical model for evaluating situational efficiency in top level handball. Int. J. Perform. Anal. Sport 13:2. doi: 10.1080/24748668.2013.11868648

[ref21] Foretić N. Rogulj N. Trninić N. (2010). The influence of situation efficiency on the result of a handball match. Sport Sci. 3, 45–51.

[ref22] Gómez-López M. Merino-Barrero J. A. Manzano-Sánchez D. Valero-Valenzuela A. (2019). A cluster analysis of high-performance handball players’ perceived motivational climate: implications on motivation, implicit beliefs of ability and intention to be physically active. Int. J. Sports Sci. Coach. 14, 541–551. doi: 10.1177/1747954119861855

[ref23] Gruiç I. Vuleta D. Milanoviç D. (2006). Performance indicators of teams at the 2003 Men’s world handball championship in Portugal. Kinesiology 38, 164–175.

[ref24] Gryko K. Bodasiński S. Bodasińska A. Zieliński J. (2018). Offensive and defensive play in handball in a 2-year world championship cycle: characteristics and tendencies. Polish J. Sport Tour. 25, 10–16. doi: 10.2478/pjst-2018-0014

[ref25] Gutiérrez Aguilar Ó. López P. (2010). Discriminant analysis between winners and losers in the asobal league 2008–2009. European Handball Federatión-Publication. Available from: http://home.eurohandball.com/ehf_files/Publikation/WP_Discriminant%20Analysis%20Winners%20Loser%20ASOBAL%202008-2009%20.pdf

[ref26] Gutiérrez Aguilar Ó. Ruiz J. L. (2013). Game performance versus competitive performance in the world championship of handball 2011. J. Hum. Kinet. 36, 137–147. doi: 10.2478/hukin-2013-0014, PMID: 23717363 PMC3661885

[ref27] Hatzimanouil D. (2019). Throwing effectiveness per throwing area and playing position among high level handball players. J. Phys. Educ. 6, 13–20. doi: 10.15640/jpesm.v6n1a2

[ref28] Kleinbaum D. G. Dietz K. Gail M. Klein M. Klein M. (2002). Logistic Regression. New York, USA: Springer.

[ref29] Konefał M. Chmura P. Kowalczuk E. Figueiredo A. J. Sarmento H. Rokita A.. (2019). Modeling of relationships between physical and technical activities and match outcome in elite German soccer players. J. Sports Med. Phys. Fitness 59, 752–759. doi: 10.23736/S0022-4707.18.08506-7, PMID: 29877676

[ref30] Korte F. Lames M. (2019). Passing network analysis of positional attack formations in handball. J. Hum. Kinet. 70, 209–221. doi: 10.2478/hukin-2019-0044, PMID: 31915491 PMC6942475

[ref32] Meletakos P. Vagenas G. Bayios I. (2011). A multivariate assessment of offensive performance indicators in Men’s handball: trends and differences in the world championships. Int. J. Perform. Anal. Sport 11, 284–294. doi: 10.1080/24748668.2011.11868548

[ref33] Milanese C. Piscitelli F. Lampis C. Zancanaro C. (2012). Effect of a competitive season on anthropometry and three-compartment body composition in female handball players. Biol. Sport 29, 199–204. doi: 10.5604/20831862.1003443 21767231

[ref34] Molfetas A. Hatzimanouil D. Papadopoulou Z. Skandalis C. Vrabas I. (2019). Analyses of technical and tactical data in attack and defense at high level handball teams. J. Phys. Educ. Sport 19, 193–200. doi: 10.7752/jpes.2019.s1029

[ref35] Oliveira T. Gómez M. Sampaio J. (2012). Effects of game location, period, and quality of opposition in elite handball performances. Percept. Mot. Skills 114, 783–794. doi: 10.2466/30.06.PMS.114.3.783-794, PMID: 22913020

[ref36] Pic M. (2018). Performance and home advantage in handball. J. Hum. Kinet. 63, 61–71. doi: 10.2478/hukin-2018-0007, PMID: 30279942 PMC6162984

[ref37] Povoas S. C. Ascensao A. A. Magalhaes J. Seabra A. F. Krustrup P. Soares J. M.. (2014). Analysis of fatigue development during elite male handball matches. J. Strength Cond. Res. 28, 2640–2648. doi: 10.1519/JSC.0000000000000424, PMID: 24552799

[ref38] Prieto J. Gómez M. Á. Sampaio J. (2015). Revisión bibliométrica de la producción científica en balonmano. Cuad. de Psicol. del Deporte 15, 145–154. doi: 10.4321/S1578-84232015000300014

[ref40] Prieto J. Gómez M. Á. Volossovitch A. Sampaio J. (2016). Effects of team timeouts on the teams’ scoring pergormance in elite handball close games. JTRM Kinesiol. 48, 115–123. doi: 10.26582/k.48.1.4

[ref41] Rogulj N. (2000). Differences in situation-related indicators of the handball game in relation to the achieved competitive results of teams at 1999 world championship in Egypt. JTRM Kinesiol. 32, 63–74.

[ref42] Rogulj N. Srhoj V. (2009). The influence of the elements of the collective attack tactics on handball match outcome. Fiz. Kult. 37, 15.

[ref43] Rogulj N. Srhoj V. Srhoj L. (2004). The contribution of collective attack tactics in differentiating handball score efficiency. Coll. Antropol. 28, 739–746. 15666606

[ref44] Rogulj N. Vuleta D. Milanovic D. Cavala M. Foretic N. (2011). The efficiency of elements of collective attack tactics in handball. Kinesiol. Slov. 17, 5–14.

[ref45] Ruiz Sánchez V. Gómez-López M. Herrera Cuadrado J. L. (2017). Observational analysis of handball shot in the counterattack phase of the national teams finalists in 2015 Qatar world handball cup. Espiral. Cuadernos del Profesorado 10, 73–79. doi: 10.25115/ecp.v10i20.1014

[ref46] Saavedra J. (2018). Handball research: state of the art. J. Hum. Kinet. 63, 5–8. doi: 10.2478/hukin-2018-0001, PMID: 30279936 PMC6162973

[ref47] Saavedra J. Pic M. Jimenez F. Lozano D. Kristjánsdóttir H. (2019). Relationship between game-related statistics in elite men’s beach handball and the final result: a classification tree approach. Int. J. Perform. Anal. Sport 19, 584–594. doi: 10.1080/24748668.2019.1642040

[ref48] Sarmento H. Bradley P. Anguera M. T. Polido T. Resende R. Campanico J. (2016). Quantifying the offensive sequences that result in goals in elite futsal matches. J. Sports Sci. 34, 621–629. doi: 10.1080/02640414.2015.1066024 26183125

[ref49] Sarmento H. Clemente F. M. Araújo D. Davids K. McRobert A. Figueiredo A. (2018a). What performance analysts need to know about research trends in association football (2012–2016): a systematic review. Sports Med. 48, 799–836. doi: 10.1007/s40279-017-0836-6 29243038

[ref50] Sarmento H. Figueiredo A. Lago-Peñas C. Milanovic Z. Barbosa A. Tadeu P.. (2018b). Influence of tactical and situational variables on offensive sequences during elite football matches. J. Strength Cond. Res. 32, 2331–2339. doi: 10.1519/JSC.0000000000002147 28737587

[ref51] Sarmento H. Marques A. Martins J. Anguera T. Campaniço J. Leitão J. (2011). Playing tactics in the English premier league, Spain’s La Liga and Italy’s Serie A. Br. J. Sports Med. 45, A6–A7. doi: 10.1136/bjsports-2011-090606.20

[ref52] Schinke R. J. Stambulova N. B. Si G. Moore Z. (2018). International society of sport psychology position stand: athletes’ mental health, performance, and development. Int. J. Sport. Exerc. Psychol. 16, 622–639. doi: 10.1080/1612197X.2017.1295557

[ref53] Skarbalius A. Krusinskiene R. (2003). Handball match analysis: computerized notation system. Int. J. Comput. Sci. Sport 2:136.

[ref54] Skarbalius A. Pukėnas K. Vidūnaitė G. (2013). Sport perfomance profile in men’s european modern handball: discriminant analysis between winners and losers. J. Phys. Educ. Sport 90, 44–54. doi: 10.33607/BJSHS.V3190.168

[ref55] Srhoj V. Rogulj N. Katić R. (2001). Influence of the attack end conduction on match result in handball. Coll. Antropol. 25, 611–617., PMID: 11811292

[ref56] Teles N. Volossovitch A. (2015). Influência das variáveis contextuais no desempenho das equipes nos últimos 10 minutos do jogo de handebol. Rev. Bras. Educ. Fís. Esporte 29, 177–187. doi: 10.1590/1807-55092015000200177

[ref57] Volossovitch A. Ferreira A. P. (2003). Gonçalves, I. The use of binominal logistic regression in performance analysis in handball. Int. J. Comput. Sci. Sport 2:145.

[ref58] Vuleta D. Milanović D. Gruić I. Ohnjec K. (2005). “Influence of the goals scored on final outcomes of matches of the 2003 World Handball Championships for men in Portugal,” in Dragan Milanovic & Franjo Prot. Proceedings book of 4th International Scientific Conference on Kinesiology, Science and Proffesion - Challenge for the future, 470–474.

[ref59] Vuleta D. Sporiš G. Purgar B. Herceg Z. Milanović Z. (2012). Influence of attacking efficiency on the outcome of handball matches in the preliminary round of men’s Olimpic games 2008. Sport Sci. 5:7.

[ref60] Wagner H. Finkenzeller T. Würth S. Von Duvillard S. P. (2014). Individual and team performance in team-handball: a review. J. Sports Sci. Med. 13:808. 25435773 PMC4234950

[ref61] Zapardiel J. C. Vila H. Manchado C. Rivilla-García J. Ferragut C. Van den Tillaar R. (2019). Effect of opposition and effectiveness of throwing 984 from first and second line in male elite handball during competition. Kinesiol. Slov. 25, 35–44.

